# Factors Affecting Recurrence and Survival for Patients with High-Risk Stage II Melanoma

**DOI:** 10.1245/s10434-023-14724-5

**Published:** 2023-12-29

**Authors:** Aikaterini Dedeilia, Thinzar Lwin, Siming Li, Giuseppe Tarantino, Sasha Tunsiricharoengul, Aleigha Lawless, Tatyana Sharova, David Liu, Genevieve M. Boland, Sonia Cohen

**Affiliations:** 1 Division of Gastrointestinal and Oncologic Surgery, Department of Surgery, Massachusetts General Hospital, Boston, MA USA; 2 Harvard Medical School, Boston, MA USA; 3 Broad Institute of Massachusetts Institute of Technology and Harvard, Cambridge, MA USA; 4 Division of Surgical Oncology, City of Hope National Medical Center, Duarte, CA USA; 5 Department of Medical Oncology, Center for Immuno-Oncology, Dana-Farber Cancer Institute, Boston, MA USA; 6 Harvard University, Boston, MA USA; 7 Cancer Center, Massachusetts General Hospital, Boston, MA USA

**Keywords:** Melanoma, Stage II, Prognostic factors, Genetic analysis, KIT, CDH1

## Abstract

**Background:**

In the current era of effective adjuvant therapies and de-escalation of surgery, distinguishing which patients with high-risk stage II melanoma are at increased risk of recurrence after excision of the primary lesion is essential to determining appropriate treatment and surveillance plans.

**Methods:**

A single-center retrospective study analyzed patients with stage IIB or IIC melanoma. Demographic and tumor data were collected, and genomic analysis of formalin-fixed, paraffin-embedded tissue samples was performed via an internal next-generation sequencing (NGS) platform (SNaPshot). The end points examined were relapse-free survival (RFS), distant metastasis-free survival (DMFS), overall survival (OS), and melanoma-specific survival (MSS). Uni- and multivariable Cox regressions were performed to calculate the hazard ratios.

**Results:**

The study included 92 patients with a median age of 69 years and a male/female ratio of 2:1. A Breslow depth greater than 4 mm, a higher mitotic rate, an advanced T stage, and a KIT mutation had a negative impact on RFS. A primary lesion in the head and neck, a mitotic rate exceeding 10 mitoses per mm^2^, a CDH1 mutation, or a KIT mutation was significantly associated with a shorter DMFS. Overall survival was significantly lower with older age at diagnosis and a higher mitotic rate. An older age at diagnosis also had a negative impact on MSS.

**Conclusion:**

Traditional histopathologic factors and specific tumor mutations displayed a significant correlation with disease recurrence and survival for patients with high-risk stage II melanoma. This study supported the use of genomic testing of high-risk stage II melanomas for prognostic prediction and risk stratification.

**Supplementary Information:**

The online version contains supplementary material available at 10.1245/s10434-023-14724-5.

With an estimated 97,000 new cases and 9000 deaths in 2023, melanoma remains the fifth most common cancer in the United States.^1^ The introduction of effective systemic therapies for the treatment of advanced melanoma has radically improved outcomes for patients with metastatic disease and has led to expanded use of both targeted therapy and immune checkpoint blockade in neoadjuvant and adjuvant settings. Because patients with resectable melanoma have the potential to attain a cure with surgery alone, there is interest in a biologically informed approach to the selection of patients for active surveillance and/or adjuvant therapy. As the field advances, understanding how best to predict recurrence in melanoma will help us limit risk, maximize benefits, and minimize cost in the multidisciplinary care of patients with melanoma.

Histopathologic characteristics such as the depth of tumor invasion, the presence of ulceration (T-stage), and the presence of local (N stage) or distant metastases (M stage) are used to stage patients with primary cutaneous melanomas and guide clinical decisions and treatment accordingly.^2,3^ The current standard of care for tumors with a Breslow depth greater than 0.8 mm or ulceration without clinical evidence of metastatic disease includes surgical excision of the primary tumor with a wide local excision (WLE) and nodal sampling via sentinel lymph node biopsy (SLNB).^4^

The eighth edition of the American Joint Committee on Cancer (AJCC) criteria uses histopathologic features to define patients without nodal metastases but with high-risk tumor features (e.g., Breslow depth >4 mm or an ulcerated tumor with a Breslow depth >2.1 mm) as having high-risk stage II disease (IIB or IIC). Early stage III disease (IIIA) is defined by the presence of clinically occult lymph node metastases in the context of a primary melanoma with a Breslow depth of less than 1 or 2 mm as long as no ulceration is seen.^3^ Interestingly, patients with stage IIIA melanomas have a higher probability of 5- and 10-year MSS (93 % and 88 %, respectively) than patients with stage IIB (87 % and 82 %) or IIC (82 % and 75 %) melanomas, suggesting that high-risk primary tumor features may be biologic drivers of recurrence and survival.^2^ Both targeted therapies and checkpoint blockade have been approved for use in the stage III adjuvant setting for patients with melanoma, and adjuvant anti-PD1 currently is approved for patients with stage IIB or IIC melanoma.^5^ However, these therapies can carry substantial risks, reinforcing the need for prognostic tools to help guide surveillance and treatment decisions.

The use of genetic factors as prognostic markers has become increasingly relevant for multiple cancers,^6^ and is even applied in routine clinical practice to identify the best treatment path for numerous malignancies.^7^ With this in mind, together with the opportunity to analyze the mutational status of primary tumors with panel gene testing (SNaPshot),^8–11^ we set out to explore the primary histopathologic and genetic factors that might have an impact on the recurrence and survival of patients with high-risk stage II melanomas.

## Methods

### Patient Selection

We performed a retrospective analysis of patients with stage IIB or IIC melanoma according to the eighth American Joint Committee on Cancer (AJCC) criteria. The patients were selected from a large melanoma cohort study and tumor biobank maintained at Massachusetts General Hospital for more than 13 years (2009–2022), and the study was approved by the Institutional Review Board. Informed consent was obtained from all patients.

### Inclusion and Exclusion Criteria

The study enrolled all patients with stage IIB or IIC melanoma according to the eighth edition of the AJCC criteria. By definition, patients with IIB or IIC melanoma had no nodal or distant metastasis (N0M0) or micrometastases, and their T stage was either T3b (Breslow depth 2–4 mm and ulcerated) or T4a (>4 mm and non-ulcerated) for IIB melanoma, and T4b (>4 mm and ulcerated) for IIC melanoma.^2^ The histopathologic types of cutaneous melanomas included were superficial spreading, nodular, spitzoid, desmoplastic, or any combination of these categories. Patients were excluded if the lesion was identified as acral lentiginous melanoma in the pathologic report due to the distinctive molecular profile and difference in natural history and prognosis of acral lentiginous melanomas.^12,13^ Mucosal lentiginous melanomas also were excluded for the same reasons.^14,15^ Previous diagnoses of melanoma *in situ,* basal cell carcinoma, or squamous cell carcinoma did not exclude patients from the retrospective study. The same was true for patients with a previous melanoma occurrence deemed unrelated to the currently studied high-risk stage II primary melanoma lesion.

As a standard practice at our institution, all tumors with stage T3b-T4b melanoma undergo wide local excision (WLE) and sentinel lymph node biopsy (SLNB). As a result, most of the patients included in our study had a histopathologically proven negative N status. However, if SLNB could not be performed but computed tomography (CT) or clinical examination demonstrated no lymphadenopathy, the patient was included in the analysis. These criteria resulted in seven patients in the cohort who did not undergo SLNB. The conclusions of the analyses presented later were unchanged after exclusion of these seven patients from the larger analysis.

Because our end points included recurrence and progression, patients who received adjuvant therapy after WLE of the primary lesion were excluded from the initial retrospective analysis. However, the entire cohort (patients with stage IIB or IIC melanoma treated or untreated with adjuvant therapy after WLE) were included as verification of the results found in the narrower analysis. Again, the inclusion of patients with stage IIB or IIC melanoma who had received adjuvant therapy did not alter the conclusions presented later.

### Data Extraction

For each patient, we extracted general demographic information, histopathologic reports from the primary biopsy and WLE, additional treatments, relapses of the tumor, and reports of genetic variations of each tumor assessed. Tumor genomic analysis of formalin-fixed, paraffin-embedded tissue had been performed for clinical purposes using SNaPshot multiplexed polymerase chain reaction (PCR) assay through the MGH Center for Integrated Diagnostics.^8–11^ The current version of the SNaPshot assay assesses single nucleotide variants and insertions or deletions in more than 100 known cancer genes. The medical record review and data extraction occurred from September 2022 through April 2023, and statistical analysis occurred in May 2023.

The clinical data extracted were name, date of birth, gender, race, age at diagnosis of the primary tumor, family history of cancer, location of primary tumor, date of biopsy, histopathologic type (superficial spreading melanoma [SSM], nodular melanoma [NM], spitzoid melanoma, desmoplastic melanoma, unknown), Breslow thickness, ulceration present, mitotic rate, lymphovascular invasion, tumor-infiltration lymphocytes, wide local excision (WLE) performed, date of WLE, status of lymph nodes assessed from the WLE, T status, tumor-node-metastasis (TNM) (by the 8th AJCC criteria) status, adjuvant therapy after excision of the primary lesion, start date and type of adjuvant therapy, delta from primary WLE to adjuvant therapy, date of recurrence if one occurred, type of recurrence (local or distant), date and type of systemic therapy after the relapse if one occurred, whether treatment was adjuvant or systemic therapy for unresectable metastasis, survival status, and date of death if it occurred. The specific mutations found in the most commonly mutated genes were also assessed.

The dates provided were used to calculate the following primary end points: RFS, DMFS, OS, and MSS from the date of WLE. For the patients without any relapse, distant metastasis, or death before the data extraction date, the date of the last follow-up visit was used as the final time point. All the patients who died of other causes unrelated to melanoma were censored for the MSS.

### Statistical Analysis

The data extraction was followed by statistical analysis using IBM SPSS Statistics for Windows, version 28.0.0.0 (IBM Corp, Armonk, NY, USA) and R (R Core Team, Vienna, Austria, 2013). The Kolmogorov-Smirnov test was used to assess the normal distribution of continuous variables, and statistical significance was set at a *p* value lower than 0.05. The mean ± standard deviation were determined for continuous variables with a normal distribution. All continuous variables are reported as median (interquartile range [IQR]) if they did not follow a normal distribution and as mean ± standard deviation if they did, and all categorical and dichotomous variables are reported as frequency (*n* [%]). Three variables (age, Breslow level of tumor invasion (in mm), and mutations found per tumor) were calculated as continuous variables, but also dichotomized into two categories each (age, >65 vs <65 years; Breslow thickness, 2–4 vs >4 mm, and mutation group of 1–4 vs ≥5 mutated genes).

The Cox regression hazards model was implemented to assess the effect of each histopathologic or genetic factor on the end points of the study (RFS, DMFS, OS, MSS). To determine their synergistic and real effect, all the statistically significant values from the univariate analysis were consecutively included in the multivariable Cox regression. Forest plots were created to depict the hazard ratios (HRs) for each RFS, DMFS, OS and MSS factor.

## Results

**Fig. 1:**
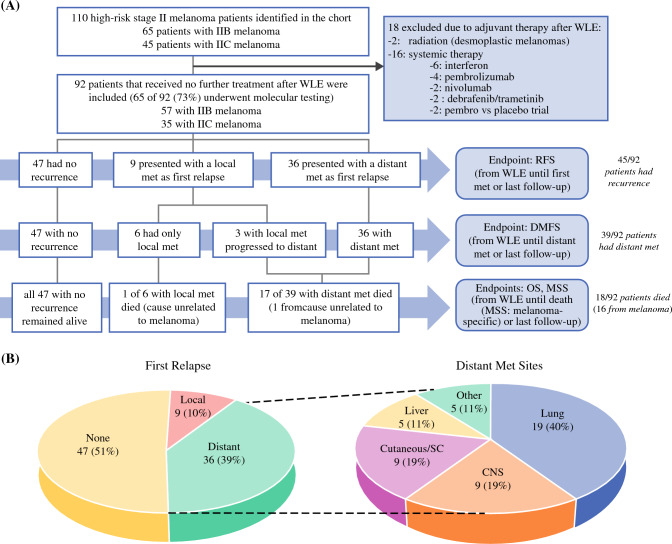
Patient flow chart, end points, and pie charts of relapses. **A** Flow chart and end points. A total of 110 patients with an initial diagnosis of stage IIB or IIC melanoma were identified from the 14-year cohort kept prospectively at MGH Cancer Center. After clinical data review and exclusion of 18 patients due to their reception of adjuvant therapy after the wide local excision (WLE) of the primary lesion, the remaining 92 patients were included in the retrospective analysis. **B** Relapse pie charts. Most patients presented with a distant metastasis as the first recurrence, with the pulmonary system the most common site of distant metastasis. WLE, wide-local excision; RFS, relapse-free survival; DMFS, distant metastasis-free survival; OS, overall survival; MSS, melanoma-specific survival; met, metastasis; CNS, central nervous system; SC, subcutaneous

From the cohort maintained at our institution, 110 patients (45 patients with stage IIB melanoma and 65 patients with IIC) were identified. Of the 110 patients, 18 had received adjuvant treatment after the primary lesion, including 2 patients enrolled in the KEYNOTE-716 trial (pembrolizumab vs placebo), 2 patients who received pembrolizumab after the Food and Drug Administration (FDA) approval (all in 2022), and 14 patients who received treatment based on individual provider practice patterns. After exclusion of these 18 patients from the initial cohort, 92 patients remained (57 with stage IIB and 35 with IIC melanoma). The median age at diagnosis was 69 years (IQR, 60–77 years) and the male:female ratio was 2:1. All demographic and histopathologic characteristics of the patients are summarized in Table 1. The patient flow chart, study end points, and recurrence pie charts are shown in Fig. 1.

**Table 1:** Patient characteristics and clinical outcomes

			*n* (%)/median (IQR)
*A. Demographic characteristics n (%)*			
Total	92 (100)	Race	
		White/non-Hispanic	83 (90.2)
Median age at diagnosis: years (IQR>	69 (60–77)	Black or African American	1 (1.1)
Age group (years)		White/ethnicity NA	3 (3.3)
≤65	35 (38.0)	Unknown	5 (5.4)
>65	57 (62.0)	Family history of malignancies	
Gender		Yes, including melanoma	16 (17.4)
Male	61 (66.3)	Yes, not including melanoma	34 (37)
Female	31 (33.7)	No	34 (37)
Other	0 (0)	Unknown	8 (8.7)
*B. Histopathologic characteristics*			
Primary location		Lymphovascular invasion	
Head and neck	19 (20.7)	Unknown	7 (7.6)
Trunk	28 (30.4)	No	74 (80.4)
Upper extremity	24 (26.1)	Yes	11 (12)
Lower extremity	21 (22.8)	Tumor-infiltrating Lymphocytes	
Primary type		Unknown	7 (7.6)
Unknown	21 (22.8)	Absent	5 (5.4)
Spitzoid	2 (2.2)	Present, non-brisk	79 (85.9)
Desmoplastic	6 (5.4)	Present, brisk	1 (1.1)
Nodular	31 (33.7)	T Stage	
Superficial spreading	33 (35.9)	T3b	35 (38)
Breslow subgroup (mm)		T4a	23 (25)
2–4	33 (35.9)	T4b	24 (37)
>4	59 (64.1)	N stage	
Median Breslow thickness: mm (IQR)	5 (3–7)	Nx	7 (7.6)
Ulceration present		N0	85 (92.4)
No	23 (25)	TNM stage	
Yes	69 (75)	IIB	57 (62)
Mitotic rate (per mm^2^)	10 (6–17)	IIC	35 (38)
*C. Molecular characteristics*			
Genetic analysis performed	(*n* = 92)	Median mutated genes found (IQR)	7 (4–10)
No	27 (29.3)	Mutation group	(N=65)
Yes	65 (70.7)	>5 mutated genes	29 (44.6)
TMB	(N=65)	0–4 mutated genes	36 (55.4)
Unknown	41 (63.1)	10 Most commonly mutated genes (frequency)	
Low (<15)	8 (12.3)	TERT (53), NF1 (25), CDKN2A (22), BRAF (21), TP53 (21), NRAS (18), ARID1A (16), TP63 (15), SMARCA4 (14), BRCA2 (13)	
High (≥15)	16 (24.6)		
Median reported TMB (IQR) (*n* = 24)	19 (8–34)		
*D. Clinical outcomes*			
Adjuvant therapy after primary	(*n* = 92)	Median follow-up: months (IQR)	37 (13–52)
No	92		
Yes	0		
Recurrence until follow-up	(*n* = 92)	Median RFS: months (IQR)	22 (8–37)
None	47 (51.1)		
Local	9 (9.8)		
Distant	36 (39.1)		
Progression after local recurrence	(*n* = 11)	Median DMFS: months (IQR)	24 (8–40)
No	7		
Distant	4		
Systemic therapy after relapse	(*n* = 44)		
No	3 (6.8)		
Yes	41 (93.2)		
Patient survival until follow up	(N=92)	Median OS: months (IQR)	37 (13–52)
Yes	74 (80.4)		
No	18 (19.6)		

IQR, interquartile range; NA, ; TNM, tumor-node-metastasis; TMB, tumor mutation burden; RFS, relapse-free survival; DMFS, distant metastasis-free survival; OS, overall survival; NA, not available

The median follow-up period was 37 months (IQR, 13–52 months) for all the patients, the median RFS was 22 months (IQR, 8–37 months), and the median DMFS was 24 months (IQR, 8–40 months) for the 92 patients. At the time of the last follow-up visit, 39.1 % of the patients had progressed to distant metastasis, 9.8 % had progressed to local metastasis, and 51.1 % had no recurrence or progression after WLE of the primary lesion.

Before the date of the data extraction 19.6 % of the patients had died, whereas 80.4 % were still alive. Genetic analysis was available for 65 (70.7 %) of the 92 patients. Importantly, the subset of patients for whom genetic analysis (SNaPshot assay) had been performed did not differ from those who had no genetic analysis performed as part of their standard clinical care (Table S1). Significant molecular characteristics and clinical outcomes are summarized in Table 1.

We performed uni- and multivariable Cox regressions to identify histopathologic and genetic mutations that affect RFS, DMFS, OS, and MSS. The following analyses investigated 92 patients for histopathologic and clinical factors and 65 patients for genetic factors. The analyses included all histopathologic factors in the 92 patients and all genes with mutations identified in at least 2 of the 65 patients (70 genes).

The univariate analysis of RFS demonstrated that it was negatively impacted by a Breslow depth greater than 4 mm (vs 0–4 mm; *p* = 0.027), a higher mitotic rate (*p* = 0.007), a mitotic rate greater than 10 mitoses per mm^2^ (*p* = 0.004), T4a stage (vs T3b; *p* = 0.017), IIC TNM stage (*p* = 0.019), NF1 mutation (*p* = 0.022), and KIT mutation (*p* = 0.005). The multivariable analysis showed that the mitotic rate and KIT mutation independently shortened the RFS. Notably, KIT mutation remained significant in the multivariable analysis (*p* = 0.009).

The univariate analysis for DMFS showed that a primary lesion in the head and neck (*p* = 0.031), a mitotic rate greater than 10 mitoses per mm^2^ (*p* = 0.026), and CDH1 mutation (*p* = 0.023) or KIT mutation (*p* < 0.001) were significantly associated with a shorter DMFS, and the same variables remained statistically significant in the multivariable analysis (all with a *p* < 0.01), meaning that a mitotic rate greater than 10 mitoses per mm^2^, CDH1 mutation, or KIT mutation correlated with an earlier distant metastasis.

The OS from the date of WLE was significantly lower with an older age at diagnosis (*p* = 0.023), a higher mitotic rate (*p* = 0.041), a mitotic rate of greater than 10 mitoses per mm^2^ (*p* = 0.025), a higher TNM stage (IIC vs IIB, *p* = 0.048), and a primary location in the trunk of the body (*p* = 0.044). In the multivariable analysis, only the age at diagnosis (*p* = 0.014) and the mitotic rate (*p* = 0.022) remained significantly associated with a shorter OS.

Finally, uni- and multivariable analyses were performed for MSS. In the univariate analysis, the age at diagnosis (*p* = 0.021) and a mitotic rate greater than 10 mitoses per mm^2^ (*p* = 0.05) were significantly associated with a worse MSS. The same significance remained in the multivariable analysis (*p* = 0.007 and 0.021, respectively), suggesting that an increase in each of these two factors was independently related to a higher risk of melanoma-specific death. The univariate Cox regressions for all the statistically significant factors affecting these four end points together with the results from the subsequent multivariable analyses are shown in Table 2.

**Table 2:** Cox regressions for factors affecting RFS, DMFS, OS, and MSS (only statistically significant results shown)^a^

	Univariate analyses					Multivariable analyses				
	HR	Lower 95 % CI	Higher 95 % CI	*p* Value	Sig	HR	Lower 95 % CI	Higher 95 % CI	*p* Value	Sig
***A. RFS***										
Breslow subgroup (>4 mm)	2.22	1.09	4.50	**0.027**	*	2.04	0.88	4.70	0.099	NS
Mitotic Rate (per mm^2^)	1.05	1.01	1.08	**0.007**	**	1.04	1.00	1.09	**0.049**	*
Mitotic rate Subgroup (>10)	2.50	1.33	4.69	**0.004**	**	NA				
T stage										
T3b (ref)	1.00	1.00	1.00							
T4a	1.70	0.76	3.83	0.198	NS	NA				
T4b	2.47	1.18	5.18	**0.017**	*	NA				
TNM stage	2.06	1.13	3.77	**0.019**	*	NA				
NF1	0.41	0.19	0.88	**0.022**	*	0.53	0.24	1.23	0.108	NS
KIT	5.68	1.67	19.29	**0.005**	**	6.01	1.56	23.1	**0.009**	**
***B. DMFS***										
Primary location										
Lower Extr (ref)	0.00	0.00	0.00			0.00	0.00	0.00		
Upper Extr	1.08	0.37	3.15	0.892	NS	0.94	0.37	2.41	0.895	NS
Trunk	1.27	0.56	2.89	0.570	NS	1.00	0.52	1.94	0.999	NS
Head and neck	2.08	1.07	4.05	**0.031**	*	2.88	1.41	5.87	**0.004**	**
Mitotic rate Subgroup (>10)	2.10	1.09	4.04	**0.026**	*	4.11	1.60	10.57	**0.003**	**
Lymphovascular invasion	2.28	0.97	5.34	0.058	†	NA				
KIT	9.21	2.58	32.92	**<0.001**	***	13.07	3.04	56.10	**<0.001**	***
CDH1	3.56	1.19	10.63	**0.023**	*	6.16	1.82	20.85	**0.003**	**
***C. OS***										
Age at diagnosis	1.06	1.01	1.11	**0.023**	*	1.08	1.02	1.15	**0.014**	*
Mitotic rate (per mm^2^)	1.06	1.00	1.12	**0.041**	*	1.09	1.01	1.16	**0.022**	*
Mitotic rate Subgroup (>10)	3.59	1.18	10.95	**0.025**	*	NA				
TNM stage	2.61	1.01	6.74	**0.048**	*	2.41	0.90	6.46	0.080	NS
Primary location										
Lower Extr (ref)	1.00	1.00	1.00			1.00	1.00	1.00		
Upper Extr	3.74	0.41	33.86	0.241	NS	3.76	0.38	36.97	0.257	NS
Trunk	4.00	1.04	15.41	**0.044**	*	3.51	0.90	13.74	0.072	NS
Head and neck	1.80	0.59	5.49	0.305	NS	1.58	0.46	5.37	0.467	NS
***D. MSS***										
Age at diagnosis	1.06	1.01	1.12	**0.021**	*	1.08	1.021	1.14	**0.007**	*
Mitotic rate Subgroup (>10)	3.05	1	9.49	**0.049**	*	1.07	1.01	1.14	**0.021**	*

Bold values indicate statistical significance (*p* values < 0.05)

RFS, relapse-free survival, DMFS, distant metastasis-free survival; OS, overall survival; MSS, melanoma-specific survival; HR, hazard ratio; CI, confidence interval; Sig, significance; NS, nonsignificant; NA, not applicable; TNM, tumor-node-metastasis; Extr, extremity; NA, not available

^**a**^Except for histopathologic factors, KIT and CDH1 mutations were associated with an earlier recurrence,specifically an earlier distant recurrence. The analysis included 92 patients for histopathologic and clinical factors and 65 patients for genetic factors (i.e., NF1, KIT, CDH1).

^*^p = 0.01–0.05; **p = 0.001–0.01; ***p < 0.001; †*p* = 0.05–0.06; NS, *p* > 0.05

**Fig. 2:**
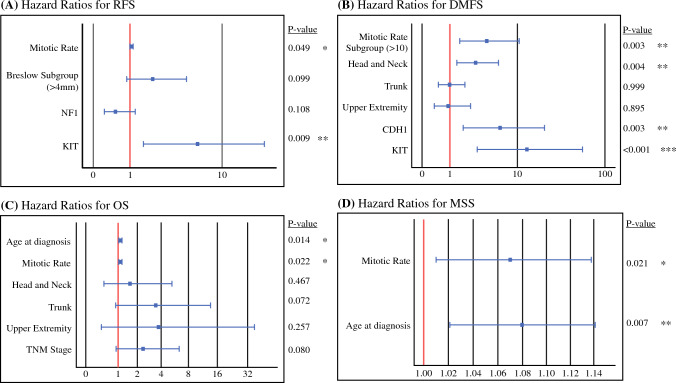
Forest plots of Cox regression hazard ratios for RFS, DMFS, OS, and MSS. Only the multivariable analyses are shown. Except for histopathologic factors, KIT and CDH1 mutations were associated with an earlier recurrence, specifically an earlier distant recurrence. The analysis included 92 patients for histopathological and clinical factors and 65 patients for genetic factors (i.e., NF1, KIT, CDH1). *p = 0.01–0.05, **p = 0.001–0.01, ***p < 0.001. RFS, relapse-free survival; DMFS, distant metastasis-free survival; OS, overall survival; MSS, melanoma-specific survival

**Fig. 3:**
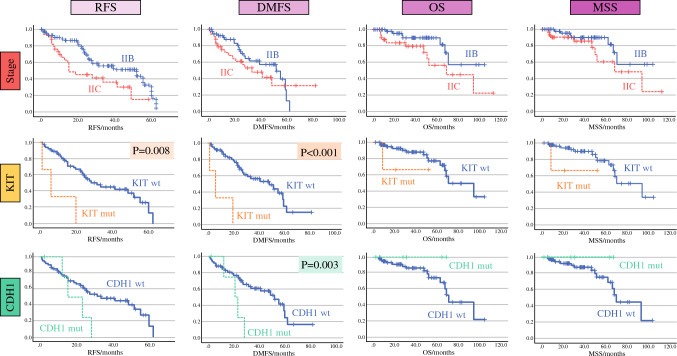
Kaplan-Meier curves of RFS, DMFS, OS and MSS based on tumor-node-metastasis (TNM) stage, KIT, and CDH1. In each Kaplan-Meier curve with no *p* value identified, the *p* value was higher than 0.05. The KIT mutation was associated with an earlier recurrence, and the CDH1 (*n* = 5) and KIT (*n* = 3) mutations were independently associated with an earlier distant recurrence. The analysis included 92 patients for histopathologic and clinical factors and 65 patients for genetic factors (i.e., NF1, KIT, CDH1). RFS, relapse-free survival; DMFS, distant metastasis-free survival; OS, overall survival; MSS, melanoma-specific survival

Additionally, forest plots of the hazard ratios of the variables included in the multivariable analyses for RFS, DMFS, and OS, and MSS are shown in Fig. 2. Kaplan-Meier curves for the four end points according to TNM stage, KIT status, and CDH1 status are presented in Fig. 3.

**Fig. 4:**
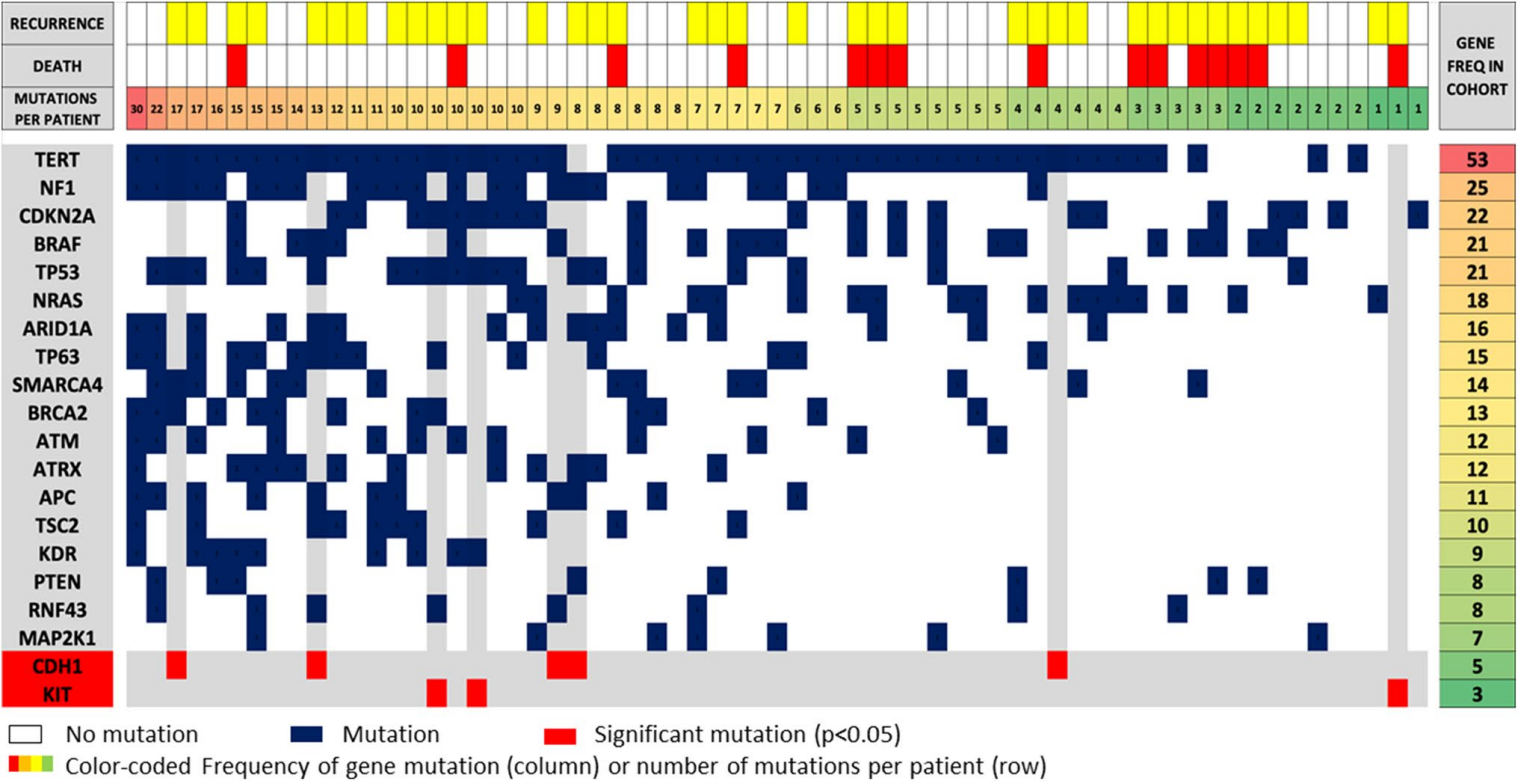
Heat map of the 20 most commonly mutated genes, identified with SNaPshot. Each column of the heat map represents one of the 65 patients for whom molecular NGS PCR data were available, and each row represents the 18 most commonly mutated genes identified from the SNaPshot reports and the two genes that were statistically significant (CDH1 and KIT). Both the columns and rows are sorted from largest to smallest moving from left to right and from top to bottom, respectively, and are supported by a color-coded row and column with their frequency in the analysis. The rows of the genes associated with a statistically significant RFS or DMFS (KIT and CDH1) and the rows of the patients who harbored mutations in these two genes are distinguished by a gray line. In the heat map, the genetic identity of the patients who harbored the significant mutations can easily be distinguished (e.g., no BRAF mutations are seen in tumors with KIT mutations, and similarly, no NRAS mutations are seen in the tumors with KIT or CDH1 mutations). NGS, next-generation sequencing; PCR, polymerase chain reaction; RFS, relapse-free survival; DMFS, distant metastasis-free survival

Of the 130+ genes identified with the SNaPshot assay, 84 were mutated in at least one patient, and 70 were mutated in at least two patients. For 55.6 % of the patients, SNaPshot analysis identified 5 or more mutated genes of interest, whereas for 54.6 % of the patients, SNaPshot analysis showed 0 to 4 mutated genes of interest. The 20 most commonly mutated genes and their frequencies are shown in Table 1, and the heat map in Fig. 4 depicts the 18 most commonly associated genes, together with the 2 statistically significant genes (KIT and CDH1). In 3 (5 %) of 65 patients KIT was mutated, and in 5 (8 %) of 65 patients CDH1 was mutated.

Tumor mutational burden (TMB) was included in a subset of SNaPshot assay reports. In this subset, 24.6 % of the patients had a high TMB (≥15 mutations), 12.3 % had a low TMB (0–14 mutations), and 63.1 % had no TMB reported. The median for those patients with a reported TMB value was 19 (IQR, 8–34). The total number of mutations among the 130 genes included in the SNaPshot testing also was calculated as an approximate alternative and used as a potential prognostic factor in the univariate Cox regressions. Neither the number of mutations identified nor the TMB value reported affected the outcomes in this cohort to a statistically significant degree.

The larger group of 110 patients identified in the cohort (including those receiving and those not-receiving adjuvant therapy) also was analyzed with Cox regressions to assess the consistency of the aforementioned results in the less refined cohort. In this larger group, 80 patients (70 %) had undergone molecular testing. A focus on the genetic mutations showed that the KIT mutation remained associated with a shorter RFS (*p* = 0.002) and also was statistically significant in the multivariable analysis (*p* = 0.001). The same was true for the KIT and CDH1 mutations affecting DMFS because both were statistically significant in the univariate analyses (*p* = 0.001 and 0.012, respectively), and their significance was preserved in the multivariable analysis (*p* = 0.013 and 0.013, respectively). No additional gene mutations were identified as statistically significant in this larger cohort.

## Discussion

Effective systemic treatments for melanoma have changed the landscape of multidisciplinary melanoma care. However, even within the same AJCC stage group, the risks of recurrence and survival for individual patients differ dramatically. Accurate prediction of outcomes for an individual patient is necessary for individualization of melanoma care. For patients with high-risk stage II melanomas, among whom approximately 20 % will have died of the disease at 10 years, adjuvant anti-PD1 therapy has been shown to improve RFS.^5,16^ The ability to predict outcomes with greater granularity in this histopathologic-based stage group would allow for personalized treatment decisions, limiting the risks of adjuvant therapies to those with the greatest chance of benefit and allocating higher stringency surveillance strategies to those at greatest risk of recurrence.

For that purpose, we limited the stage II cohort for our study to include only patients with high-risk stage II tumors (IIB or IIC) based on the AJCC histopathologic staging system. Of 110 identified patients, we defined a treatment-free sub-cohort of 92 patients, none of whom had received any adjuvant treatment after the WLE of their primary melanoma. Given the more recent understanding of the risk for stage IIB or IIC melanoma, most of these patients had been added to our cohort in the last 3 years before the analysis. Therefore, only 30 of the 92 patients had reached 5-year follow-up evaluation. For these patients, the total 5-year MSS was calculated at 79 %, which is comparable with the 5-year MSS calculated by the eighth AJCC criteria (82 % for IIB and 87 % for IIC), and the 5-year MSS of the adjuvant-free arm of the KEYNOTE-716 trial.^2,3,5,16^ Therefore, this cohort appears to be representative of patients with high-risk stage II melanoma based on previously published multi-institutional data.

This cohort provided the opportunity to define features of high-risk stage II melanomas not previously available in other published cohorts or databases such as The Cancer Genome Atlas (TCGA) program. For example, we were able to define patterns of recurrence in this high-risk cohort in greater detail than previously reported. We observed that most of the patients who had recurrence or progression of disease after resection of the high-risk primary melanoma presented with an initial distant metastasis (39 %) rather than a local recurrence (10 %). In the placebo arm of the KEYNOTE-716 trial (a group similarly not treated with adjuvant systemic therapy), distant metastases also were more common than local, regional, or locoregional events at first recurrence (16 % vs 11 %), and 29 % of the patients with a locoregional recurrence went on to experience distant metastases.^16^

Interestingly, the metastatic recurrences seen in our cohort represented all M stages (M1a–M1d). The most common initial site was the lungs (M1b, 40 %), followed by the brain (M1d: 19 %), dermal and/or subcutaneous (M1a, 19 %); the liver (M1c, 11 %); and all other locations (M1c, 11 %). Pie charts showing the breakdown of the local and distant recurrences are shown in Fig. 1B. These results suggest that a melanoma with high-risk primary tumor features, as seen in stage IIB and IIC tumors, can be an aggressive disease with relatively high rates of distant metastatic recurrence. This is supported by evidence discussed earlier showing that patients with IIB or IIC melanoma have worse MSS than those with limited nodal metastases but favorable primary tumor features.^3^ This supports the potential benefit of systemic pembrolizumab for stage IIB or IIC melanoma, as seen in the KEYNOTE-716 trial, and the need to define prognostic features within this high-risk group in order to accurately guide biologically informed treatment strategies.

Histopathologic features, such as Breslow tumor thickness and mitotic rate, have been used previously to inform predictions of high-risk, stage II melanoma recurrence and overall survival,^17^ and the same factors were statistically significant predictors in our study. As expected, a greater Breslow depth of invasion of the primary melanoma (>4 vs 0–4 mm), a higher mitotic rate (specifically >10 mitoses vs 0–10 mitoses per mm^2^), the T stage (T4b vs T4a vs T3b), and the TNM stage (IIC vs IIB) of the primary lesion were significantly associated with shorter RFS, DMFS, and OS. The presence of lymphovascular invasion (LVI) also trended toward statistical significance for a shorter DMFS (*p* = 0.058). In the literature, LVI is shown to have an impact on clinical outcome,^18^ and the lack of statistical significance in this study may relate to sample size, which also may have had an impact on other histopathologic factors in our results. Age at diagnosis also was statistically associated with shorter OS and MSS, and this is congruent with prior publications.^19^ Additionally, the location of the primary tumor was shown to influence DMFS, with lesions on the head and neck showing a shorter time to distant metastasis than lesions in any other location, which also has been reported previously.^17^

The most noteworthy findings of our study lay in the molecular prognostic factors because we were able to define gene mutations in the cohort that predicted the recurrence risk and were suggestive of high-risk melanoma biology. In 3 (5 %) of the 65 patients with genetic data, KIT mutation was present and correlated with shorter RFS and DMFS. These KIT mutations included a Leu576Pro mutation in exon 11, a Lys642Glu mutation in exon 13, and a His697Tyr mutation in exon 14. Each of these mutations has been reported previously as a driver mutation. The tumor with the Leu576Pro (exon 11) mutation showed no other mutations in the SNaPshot analysis, consistent with a driver function. The other two tumors had nine additional mutations identified with SNaPshot.

In melanoma, KIT mutations have been historically associated with mucosal and acral melanomas^20^ and only rarely have been identified in the metastases of stage IV cutaneous melanomas of sun-damaged areas.^21^ In our cohort, a KIT mutation found in stage IIB or IIC cutaneous melanoma was associated with a higher risk for metastasis, specifically distant metastasis. These findings may suggest that KIT-mutated melanomas are more aggressive, with a higher risk of progression to distant metastasis. Interestingly, Carvajal et al.^22^ found that both the Leu576Pro and Lys642Glu mutations responded to imatinib in the metastatic melanoma setting, supporting the possibility of expanding imatinib to adjuvant application in a high-risk stage II setting in KIT-mutated melanoma.

We also identified that CDH1 (the gene that encodes E-cadherin) was mutated in 5 (8 %) of the 65 patients with SNaPshot and associated with an increased risk of an earlier distant recurrence. As with KIT mutations in our cohort, all the CDH1 mutations were single-nucleotide variants (Pro373Leu, Gly633Arg, Leu729Arg, Thr253Ile, and one splice region variant). The CDH1 gene is a known tumor-suppressor gene, and the loss of E-cadherin expression is an important indicator of metastatic potential and it is associated with tumor metastases in a wide range or tumors (gastric, breast, colorectal, thyroid, and ovarian tumors).^23^ Studies focused on melanoma have shown that normal expression of E-cadherin inhibits the invasion of melanoma cells into the dermis by downregulating invasion-related adhesion receptors and inducing apoptosis. The disruption of E-cadherin control of melanoma cells has been suggested to drive tumor melanocyte transformation, survival, and invasion.^24^ Other studies, concordant with our conclusions, have highlighted that CDH1 mutations may have a significant prognostic effect in melanoma.^25^ These particularly interesting findings prompt further research into the importance of this gene in melanoma biology.

The co-occurrence or mutual exclusivity between mutations can play a pivotal role in identifying distinct genomic phenotypes within melanoma stage groups. In our cohort, KIT mutation was mutually exclusive with BRAF, NRAS, and NF1 mutation. It has previously been shown that KIT mutations are enriched in “triple wild-type melanomas” (BRAF−, NRAS−, NF1−), which are less likely to be driven by an ultraviolet (UV) signature and carry significantly more copy-number segments and complex rearrangement events.^26–28^ The finding that the KIT mutation is enriched in this triple wild-type subgroup suggests that KIT constitutes a driver mutation in these tumors. The identification of the KIT mutation as a poor prognostic factor in high-risk stage II melanomas suggests that testing for this mutation may provide an opportunity for the adjuvant use of KIT inhibitors to treat these otherwise treatment-resistant triple wild-type tumors.

Interestingly, although telomerase reverse transcriptase (TERT) promoter mutations did not independently have a statistically significant effect on the recurrence risk and survival of the patients in our cohort, TERT-promoter mutations were identified in 83 % of the primary melanomas. Previous studies have reported that 37.9 % of primary melanomas harbored a TERT mutation,^29^ whereas they showed that, a higher percentage of TERT mutations (74 %) was identified in the metastases of these same melanomas. Our finding of a relatively high percentage of TERT mutations in primary melanomas in this cohort may reflect the high-risk nature of these stage IIB and IIC melanomas. The TERT-promoter mutations upregulate telomerase activity, allowing cancer cells to survive, and prior studies have shown TERT-mutant melanomas to have earlier recurrence and progression, as well as resistance to treatment.^29,30^

**Fig. 5:**
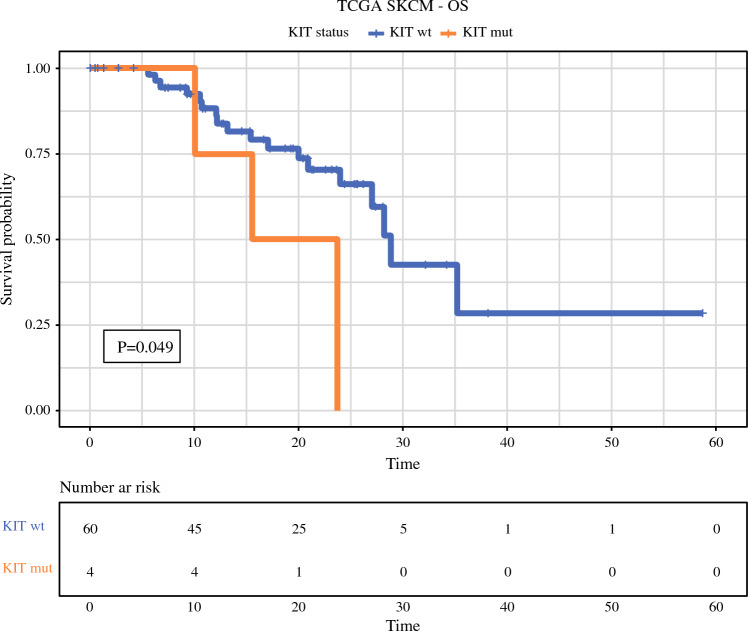
Kaplan-Meier curves of OS according to KIT mutation (TCGA data validation). In the validation data from TCGA, the patients with a KIT mutation had a shorter OS. Wt, wild-type; mut, mutated; OS, overall survival; TCGA, The Cancer Genome Atlas

Analysis of NGS data for cutaneous melanomas available in the TCGA and Genomics Evidence Neoplasia Information Exchange (GENIE) cBioportal databases supported the observations made in our cohort.^31,32^ In the TCGA database, selecting only the primary tumors that are T3b and M0 (*n* = 64), a KIT mutation was associated with a worse OS, as shown in Fig. 5. This conclusion further certifies the role of the KIT mutation as a prognostic factor. In the publicly available GENIE dataset, looking exclusively at cutaneous melanomas shows that the specific mutation rates within primary tumors were 55 % for TERT, 40 % for BRAF, 7.2 % for KIT, 2.5 % for NRAS, and 1.9 % for CDH1. Based on GENIE data, KIT mutations significantly co-occurred with CDH1 (*p* = 0.012) and TERT (*p* = 0.047) mutations and were mutually excluded with NRAS (*p* = 0.013) and BRAF (*p* = 0.039) mutations, whereas mutual exclusivity also was observed between CDH1 and NRAS mutations (*p* = 0.047) and NRAS and BRAF mutations (*p* = 0.001) in cutaneous melanomas.

Finally, TERT mutation was seen in 75 % of melanoma metastases of tumors logged in the GENIE database, with significant enrichment in metastases versus primary tumors (*p* < 0.001). These results highlight the different genetic subtypes of cutaneous melanomas and point to a hypothesis that the TERT+KIT+ melanoma subtype may be a high-risk phenotype that results in earlier recurrences.

The use of genetic characteristics as prognostic factors has been studied in multiple malignancies and has proved to have great potential in standard clinical decision-making for specific diseases and settings.^6,33–36^ Similarly, various gene signatures and expression profile tests have been proposed as effective prognostic factors of melanoma recurrence and survival.^37–42^ Instead of creating a predetermined panel to test the effect on prognostication, our study used existing platforms deployed in stage III and IV melanoma as standard testing to examine mutational status in primary lesions of high-risk stage II melanomas and associate it with clinical outcomes. None of the significant genes from our study have been evaluated or included in commercially available genetic panels, so we cannot compare our results with those of other platforms. However, our data do support a role for deeper genomic analysis for high-risk patients. Whereas most institutions routinely test high-risk patients with melanoma for actionable BRAF mutations, expansion of the routine genetic panel used may facilitate the application of risk stratification and treatment decisions based on specific mutations.

Appropriate risk stratification also could guide surveillance of patients with high-risk stage II melanomas. The current National Comprehensive Cancer Network (NCCN) criteria provide broad recommendations for surveillance (e.g., clinical evaluation and complete skin check every 3 to 6 months for the first 2 years of follow-up evaluation, then every 6 to 12 months for the next 3 to 5 years for all patients with stages IIB to IV melanoma, and annually as clinically indicated). Follow-up imaging for all patients with stages IIB to IV disease is recommended every 3 to 12 months for the first 2 years, then every 6 to 12 months for the next 3 years. The European Society for Medical Oncology (ESMO) guidelines provide no consensus on the optimal follow-up and imaging schedule.

A need exists for patient-tailored recommendations because under-surveillance might lead to delayed diagnosis of disease progression and result in worse patient outcomes, whereas over-surveillance of patients who might never have recurrence can create unneeded patient anxiety and increase health care costs. Studies have shown that proper surveillance imaging can prove extremely cost-effective in melanoma because it avoids diagnostic errors and aids in the early and proper diagnosis as well as early treatment of distant disease.^43^ Additionally, in this era when a multitude of surveillance tools are being added to the toolbox of longitudinal follow-up evaluation, they can help in accurately and cost-effectively diagnosing disease recurrences earlier.^44^ Therefore, a more personalized approach to treatment and surveillance of patients with IIB or IIC melanoma could not only improve patient outcomes, but also provide a significant cost benefit for the health care system and affected patients and families.

## Study Limitations

This study was a retrospective analysis from a single tertiary institution. Therefore, selection bias could have been present due to referral patterns. Given the new adjuvant options for patients with high-risk stage II melanoma, the cohort was weighted toward patients with a shorter follow-up period, which may have had an impact on our findings. In addition, the number of patients and lack of events until the last follow-up evaluation, in addition to the small number of mutations present in the group, could potentially have led to false-negative results (type 2 errors), or even false-positive results (type 1 errors). Validation with other cohorts (e.g., the GENIE and TCGA cBioportal databases) helped to avoid type 1 errors, and type 2 errors can be assessed in further follow-up studies, which can implement this hypothesis-forming initial study.

## Study Strengths

Despite these limitations, our study had several strengths including standardized treatment algorithms for most patients and the ability to integrate standard-of-care in-house genetic analysis, which has previously been routinely performed for all patients with stage III or IV melanoma. This enabled us to study the effect of more than 100 clinically relevant genes on the RFS, DMFS, OS, and MSS of the patients with stage II melanoma and may support the application of existing genomic tests in localized melanoma patient populations.

## Conclusion

Our retrospective study assessed existing prognostic factors that may affect recurrence and melanoma-associated survival in high-risk stage II melanomas and evaluated strategies to allow biologically informed care such as escalation or de-escalation of follow-up evaluation and treatment. In addition to known histopathologic features, genomic features were integrated as prognostic factors that correlated with recurrence and survival for patients with high-risk stage II (IIB and IIC) melanomas. A KIT mutation was associated with shorter RFS, and KIT and CDH1 mutations were associated with shorter DMFS. These data are exploratory and hypothesis-generating and require larger validation in additional cohorts but offer a rationale for the implementation of existing genomic analyses used in stages III and IV melanoma and for risk stratification in high-risk stage II melanomas.

### Supplementary Information

Below is the link to the electronic supplementary material.

Supplementary file1 (DOCX 14 KB)
