# Supplementary material for: Factors Affecting Recurrence and Survival for Patients with High-Risk Stage II Melanoma
Source: Ann Surg Oncol. 2023 Dec 29;31(4):2713–26. doi: 10.1245/s10434-023-14724-5 (PMC10908640; doi:10.1245/s10434-023-14724-5)
Supplement: Supplementary file 1 — Supplementary file1 (DOCX 14 KB) [file 10434_2023_14724_MOESM1_ESM.docx]

**Supplemental Table 1: Comparison of histopathological variables of patients who underwent SNaPshot, with those who did not.**

| **Supplemental Table 1: Comparison of histopathological variables of patients who underwent SNaPshot, with those who did not.** | | | |
| --- | --- | --- | --- |
| **Histopathological variable** | **Patients with SNaPshot**  **(n=65)** | **Patients without SNaPshot**  **(n=27)** | **p-value** |
| Age at Diagnosis (mean±SD) | 67.8 (±11.8) years | 69.3 (±14.8) | 0.150 |
| Breslow Depth (mean±SD) | 7.2 (±8.5) | 5.6 (±4.4) | 0.419 |
| Mitotic Rate (mean±SD) | 12 (±8) | 10.6 (±7.4) | 0.994 |
| Ulceration (present,%) | 47/65, 72,3% | 22/27, 81.5% | 0.355 |
| Lymphovascular infiltration (present, %) | 9/61, 14.8% | 2/22, 9% | 0.427 |

**Supplemental Table 1: Comparison of histopathological characteristics of patients who underwent SNaPshot, with those who did not.** Even though SNaPshot report was not generated for all primary high-risk stage II melanomas in our cohort, statistical analysis (T-test for continuous variables and Chi-square for categorical variables) showed that there were no histopathological differences, therefore the subpopulation of patients with SNaPshot reports did not have a worse biology and prognosis. All variables in the table are reported as mean (SD) or as N (%), depending on if they are continuous or categorical.
